# Supplementary material for: High-Dimensional MR Reconstruction Integrating Subspace and Adaptive Generative Models
Source: IEEE Trans Biomed Eng. Author manuscript; Available in PMC 2025 Jun 1. (PMC11105985; doi:10.1109/TBME.2024.3358223)
Supplement: Supplementary Material [file NIHMS1985802-supplement-Supplementary_Material.pdf]

Supplementary Materials

Algorithm 1: A Summary of the Proposed Adaptation and Reconstruction Strategy

Part 1: Subject-specific GAN Adaptation

Input: Pretrained StyleGAN2: $G_{\boldsymbol{\theta}_p}(\cdot)$ (parameterized by $\boldsymbol{\theta}_p$) and reference image from the subject: $\mathbf{x}_p$

Algorithm:

(a) Fix $\boldsymbol{\theta}_p$, update the latents $\mathbf{w}_p$ by:

$$\hat{\mathbf{w}}_p = \arg\min_{\mathbf{w}_p} \left\| G_{\boldsymbol{\theta}_p}(\mathbf{w}_p) - \mathbf{x}_p \right\|_F^2, \qquad \text{(Eq. (6) in the main text)}$$

(b) Fix the latents as $\hat{\mathbf{w}}_p$, update $\boldsymbol{\theta}$ by:

$$\hat{\boldsymbol{\theta}} = \arg\min_{\boldsymbol{\theta}} \left\| G_{\boldsymbol{\theta}}(\hat{\mathbf{w}}_p) - \mathbf{x}_p \right\|_F^2 + \alpha \left\| \boldsymbol{\theta} - \boldsymbol{\theta}_p \right\|_F^2. \qquad \text{(Eq. (7) in the main text)}$$

Output: Adapted GAN representation of the reference image: $\boldsymbol{G}_{\hat{\boldsymbol{\theta}}}(\hat{\mathbf{w}}_p)$

Part 2: Adapted GAN Constrained Subspace Reconstruction

Input: Adapted GAN: $\boldsymbol{G}_{\hat{\boldsymbol{\theta}}}(\cdot)$, acquired data: $\mathbf{y}$, predetermined basis $\hat{\mathbf{V}}$, initial estimate $\hat{\mathbf{U}}^0$ and encoding operator $\mathbf{A}$

while not converged **do**

Update latent $\mathbf{w}$ by solving:

$$\{\hat{\mathbf{w}}_t^{i+1}\} = \arg\min_{\{\mathbf{w}_t\}} \sum_{t=1}^{N_t} \left\| (\hat{\mathbf{U}}^i \hat{\mathbf{V}})_t - G_{\hat{\boldsymbol{\theta}}}(\mathbf{w}_t) \right\|_F^2, \qquad \text{(Eq. (8) in the main text)}$$

Update spatial coefficients $\mathbf{U}$ by solving:

$$\hat{\mathbf{U}}^{i+1} = \arg\min_{\mathbf{U}} \left\| \mathbf{y} - \mathbf{A}(\mathbf{U}\hat{\mathbf{V}}) \right\|_2^2 + \sum_{t=1}^{N_t} \lambda_{1,t} \left\| (\mathbf{U}\hat{\mathbf{V}})_t - G_{\hat{\boldsymbol{\theta}}}\left(\hat{\mathbf{w}}_t^{i+1}\right) \right\|_F^2 + \lambda_2 R(\mathbf{U}\hat{\mathbf{V}}), \qquad \text{(Eq. (9) in the main text)}$$

$i \leftarrow i + 1$.

end

Output: The reconstructed spatial coefficients: $\hat{\mathbf{U}}$.

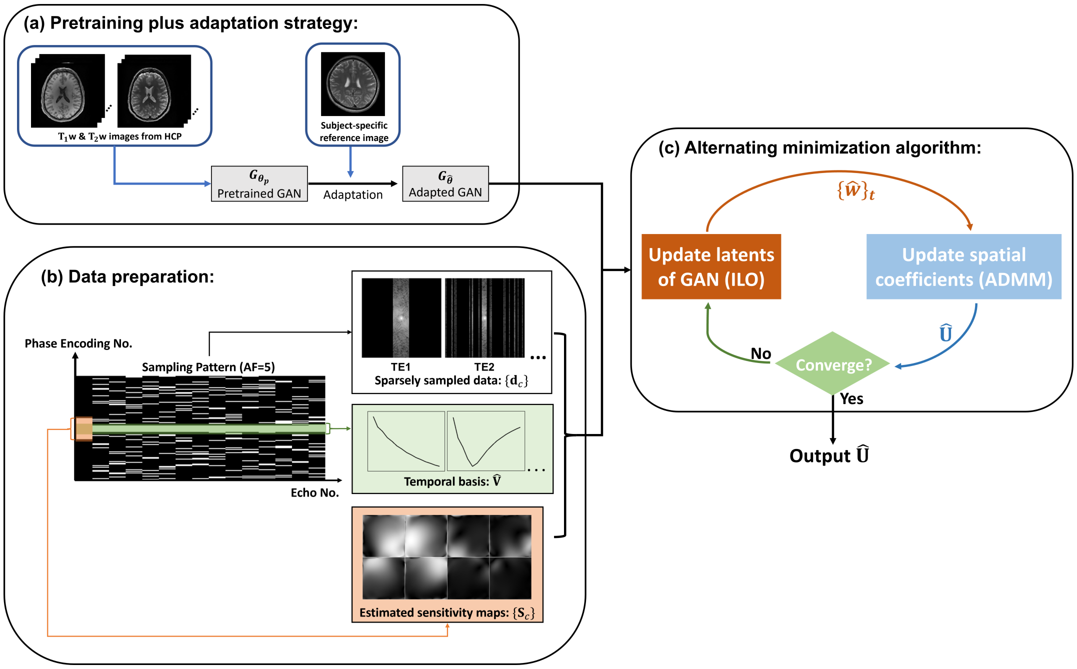

Fig. S1. An illustration of the reconstruction workflow for accelerated T_2 mapping: (a) Subject-specific network representation $G_{\hat{\theta}}$ was obtained by adapting a StyleGAN2 pretrained on HCP data and used as image-domain constraints for different contrast-weighted images; (b) Sparsely sampled data were acquired; temporal basis and sensitivity maps (first TE) were estimated from partial data; (c) An alternating minimization algorithm that updates the latents and spatial coefficients alternatively was developed to solve the optimization problem using the multichannel, sparse $\boldsymbol{k}$-space data.

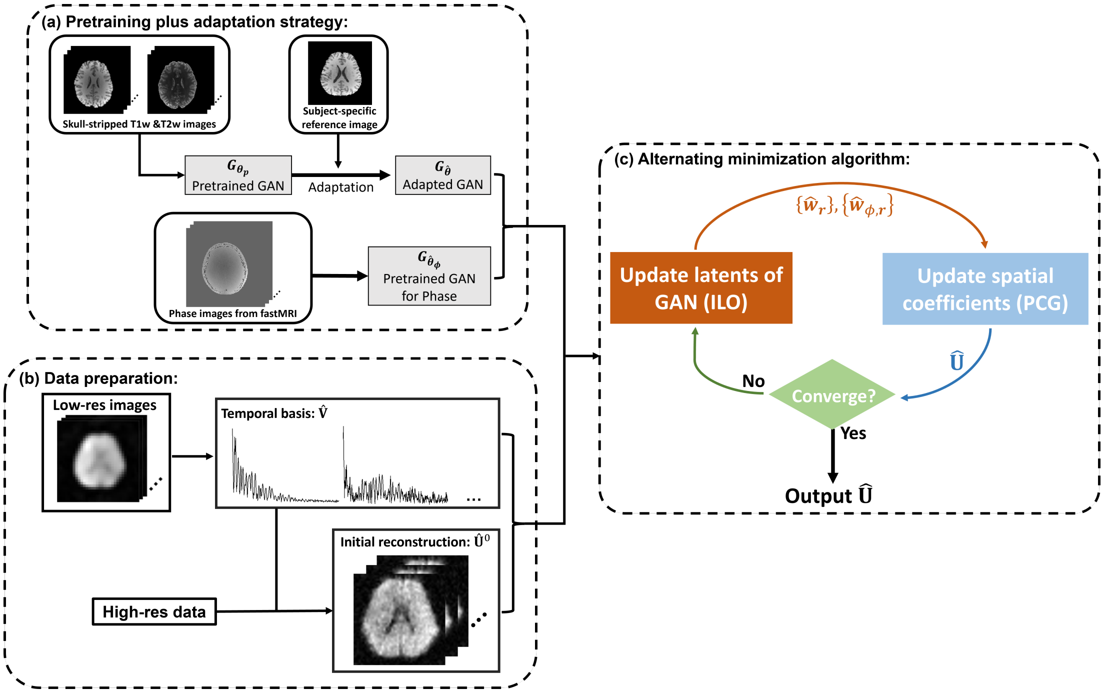

Fig. S2. An illustration of the reconstruction workflow for the high-resolution MRSI problem: (a) The same pre-training plus adaptation strategy was used, producing a constraint for the spatial coefficients. An additional phase network pretrained on NYU fastMRI data was introduced for phase constraint; (b) Temporal basis was pre-estimated from subject-specific low-resolution data (higher SNR). An initial subspace reconstruction was performed for algorithm initialization; (c) A similar alternating minimization algorithm was used to solve the reconstruction problem. Latents for magnitude and phase networks were updated alternatively during the styleGAN2 latent update step.

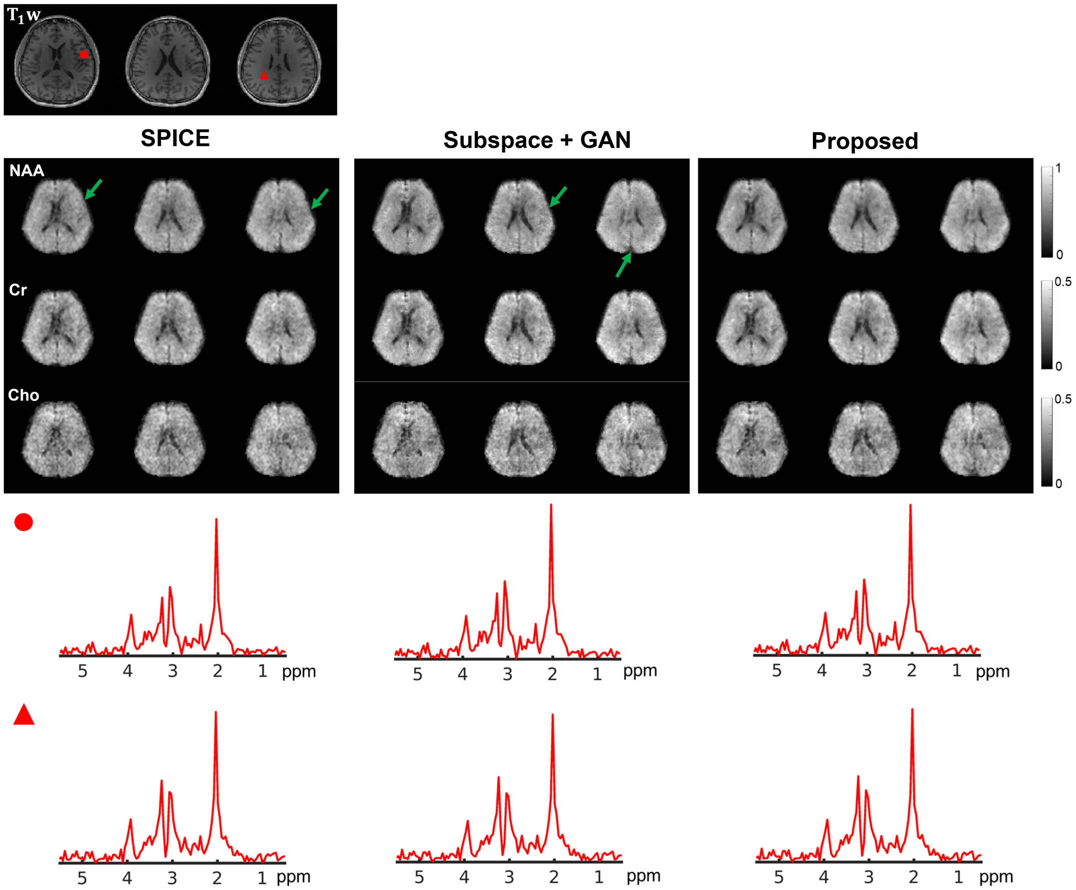

Fig. S3. Additional comparison of the proposed method to Subspace+GAN (without the edge-preserving regularization) for the same in vivo MRSI data shown in Fig. 7 in the main text: Similar contrast was observed in the results produced by Subspace+GAN and the proposed method, while the latter shows a better SNR. Similar spectral quality was observed.
